# Identification and Preservation of Intestinal Parasites Using Methylene Blue-Glycerol Mount: A New Approach to Stool Microscopy

**DOI:** 10.1155/2014/672018

**Published:** 2014-04-10

**Authors:** Vinay Khanna, Kriti Tilak, Shihnin Rasheed, Chiranjay Mukhopadhyay

**Affiliations:** Department of Microbiology, Kasturba Medical College, Manipal University, Karnataka 576104, India

## Abstract

We have tried a new approach to routine stool microscopy by using a combination of methylene blue and glycerol in wet mount preparation of fresh faecal samples for the demonstration of medically important intestinal parasites. This combination was evaluated for finding differences in the details and clarity of morphology and internal structures of parasites under low- and high-power microscopy as compared to iodine and saline mount. It was further evaluated to estimate the time taken by methylene blue-glycerol mount to dry up as compared to iodine and saline wet mount.

## 1. Introduction

Parasitic infections, caused by intestinal helminths and protozoans, account for significant burden of human disease load in the developing countries. It is estimated that around 3.5 billion people harbour parasites and 450 million are ill as a result of these infections [1]. Intestinal helminths, however, are rarely a cause of mortality. Parasites of diarrhoeal aetiology are widespread in water supplies, infecting a significant proportion of the human population in the third-world countries [2], especially across the Asian subcontinent. With an exploding population leading to overcrowding and unhygienic practices, these parasites pose a serious threat that is worsened by limited resources [3]. Over the last decade, new approaches to the diagnosis, treatment, and prevention of intestinal protozoan parasites have been developed. However, a considerable improvement in the diagnosis and treatment of intestinal helminthic infections are yet to be achieved.

Faeces are the most frequent specimens collected and examined for demonstration of parasites of the gastrointestinal tract. In a parasitology laboratory, routinely, two preparations of each specimen are usually made on each slide: one unstained preparation and another temporarily stained preparation. The saline wet mount is an unstained preparation made by using physiological saline. The advantage of saline preparation is that it helps to demonstrate the motility of trophozoites. However, it does not contribute much to the definitive diagnosis of cysts or trophozoites, because internal structures are often poorly visible. To overcome this disadvantage, several stain solutions have been used for preparation of temporarily stained wet mounts of faecal specimens. The advantages of direct wet mount include the following: (i) it is a fast, simple procedure and provides a quick answer when positive [4]; (ii) it provides an approximation of the parasitic burden [4]; (iii) it can be used with unpreserved specimens to detect the characteristic motility of trophozoites [4]; (iv) it can be used as a safeguard, as some protozoa may at times not concentrate properly because of unknown factors [5]; (v) it can detect the motile trophozoites stage of the protozoan species [6].

The classical technique described for the microscopic examination of parasites is the iodine mount method, which aids in the differentiation and identification of parasites by characteristic morphological features and details of internal structures. The method is simple to perform, quick, and inexpensive, facilitating direct visualization of parasitic ova and cyst morphology. The disadvantage of this technique is that the preparation dries within a few minutes, rendering it unreadable and unreliable to visualize live nematodal larvae [7]. Each time a fresh preparation is required to view slides for later consultation or demonstration, which consumes considerable resources and technician's valuable time. These shortcomings can be overcome by using methylene blue dye and glycerol combination. This wet mount is a simple, economical substitute to the iodine mounts and saline mounts as it lasts longer and provides better visualization and differentiation from vegetable matters and other debris in the stool, thereby facilitating any subsequent expert confirmation. Glycerol, by its hygroscopic nature, absorbs water molecules from the environment and prevents drying out of wet mounts.

## 2. Aims and Objectives

*Objective 1*. This work aims to evaluate differences in the morphological details and clarity of parasites from surrounding debris and artefacts in stool under low- and high-power microscopy.

*Objective 2*. It also aims to evaluate the time taken by methylene blue dye with different dilutions of glycerol mount to dry out at ambient tropical laboratory temperature (25 ± 2°C) compared to the time taken by control (conventional mounts without glycerol).

## 3. Material and Methods

### 3.1. Specimens

One hundred and fifty fresh unpreserved faecal specimens were collected from symptomatic patients attending tertiary care hospital from south India with history of various intestinal symptoms such as diarrhoea, abdominal pain, vomiting, and perirectal itching, over a study period of 1 year. All faecal specimens were examined using methylene blue-glycerol wet mount and compared with iodine and saline wet mounts without glycerol.

### 3.2. Preparation of Wet Mounts of Faeces

#### 3.2.1. Methylene Blue-Glycerol Wet Mount

A thick smear of faecal sample was prepared by adding a greater volume of faeces to a drop of methylene blue-glycerol solution on a microscope glass slide and placing a coverslip over the smear. Methylene blue-glycerol solution was prepared by mixing methylene blue dye separately with increasing quantities of glycerol in concentrations of 10%, 12.5%, 16.6%, 25%, and 50%.

#### 3.2.2. Saline Wet Mount and Iodine Wet Mount

Saline wet mounts and iodine wet mount were prepared by separately mixing a small volume of stool sample with a drop of physiological saline, methylene blue dye, and Lugol's iodine (diluted in 1 : 5 distilled water), respectively, on a glass slide and placing a coverslip over the smear.

### 3.3. Faecal Microscopy

Saline wet mount and iodine wet mount of specimen were first examined followed by the methylene blue-glycerol wet mounts which were mounted using solutions containing varying concentrations of glycerol. The entire wet mounts were examined initially by using a low-power (10x) objective and then again by using a high-power (40x) objective of the compound microscope.

## 4. Results and Discussion

A total of one hundred and fifty samples were observed using methylene blue-glycerol, saline, and Lugol's iodine, out of which fifty were found to be positive for various parasites. Majority of the parasites were found to be* Entamoeba histolytica* and* Strongyloides stercoralis* and one sample, contaminated with* Paramecium *spp., was taken to observe the morphological details of the organism (Table 1).

After observing various parasites by different mounting techniques, it was found that methylene blue-glycerol combination provided an excellent contrast between the parasitic structures and the artefacts as compared to saline and iodine mount. Internal structures of trophozoites, cysts, and ova were stained well by methylene blue dye, which facilitated their recognition and identification in specimens. Refractive index of glycerol is greater than that of water, which helps to demarcate the characteristic morphological features and details of internal structures, thus assisting in differentiation and identification of parasites. A similar type of study was done by Parija and Prabhakar [8] to evaluate the use of lactophenol cotton blue wet mount preparation as an alternative to routine mounts and found similar results with different parasites. We found that the preparation of lactophenol cotton blue requires more numbers of reagents, is expensive, and is more cumbersome as compared to methylene-glycerol mount.

Vignesh et al. have evaluated 0.25% iodine-glycerol wet mount preparation for the detection of intestinal parasites [9]. One of the drawbacks of iodine wet mounts is that the cysts are stained poorly with the iodine and are less retractile than unstained cysts in the saline preparation; therefore, they are difficult to visualize and can easily be missed. In contrast, cysts in methylene blue-glycerol wet mounts were stained blue which could easily be observed even during screening of stool specimens with a low-power objective. This is immensely valuable in screening the stool samples for the parasites rapidly and effortlessly. This will be particularly beneficial for unskilled students doing microscopic examinations for stool parasites. Moreover, this method was simple to perform, time saving, and inexpensive. It allowed direct visualization of parasitic ova and cyst morphology. It was possible to visualize living and motile organisms.

**Figure 1:**
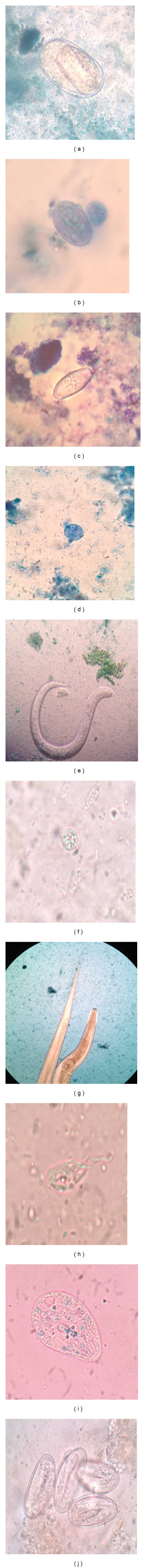
Hookworm ova (a), cyst of* Giardia lamblia* (b), oocyst of* Isospora belli* (c), trophozoites of* E. histolytica* (d),* Strongyloides stercoralis* larvae (e), oocyst of* Cyclospora *spp. (f), pinworm (g), trophozoite of* Trichomonas vaginalis* (h),* Paramecium *spp. (i), and eggs of* Enterobius vermicularis* (j) as seen on methylene blue-glycerol mount under microscope at 40x magnification.

A noted disadvantage of saline wet mounts is that colorless, nonbile-stained ova of hookworms and* Hymenolepis nana*, and so forth, are often missed due to the same backdrop in stool examinations, mostly while screening with a low-power objective. In methylene blue-glycerol wet mounts, helminthic ova were stained deep blue thus providing an excellent contrast and making them very easy to visualize even during low-power microscopic screening. Moreover, vegetable cells, mucus, and other artefacts in the stool were clearly stained in methylene blue-glycerol mount, making them distinguishable as artefacts (Figure 1).

One of the shortcomings of the methylene blue-glycerol mount is that bile-stained helminthic ova, such as ova of* A. lumbricoides* and* T. trichiura, *lose their innate brown color and are stained blue. This makes it difficult to differentiate bile-stained eggs from nonbile-stained ones. This difficulty, obviously, occurs with any wet mount preparation using temporary stains. For example, in iodine wet mounts, both bile-stained and nonbile-stained helminthic ova are stained brown. This disadvantage of methylene blue-glycerol mount can be avoided by examination of saline wet mount of the same stool sample to detect bile-stained eggs.

Glycerol provided a semipermanent preparation by acting as a preservative due to its hygroscopic nature, thus preventing early drying of the mount. It also worked as a fixative. Therefore, repeated mounting was not required. Iodine or saline mount, which was taken as a control, dried in less than 10 minutes, whereas methylene blue-glycerol mount lasted much longer without drying. It started drying only after 6 hours and could be retained for up to 5 days under different dilutions. The time taken by the methylene blue-glycerol mount to dry up varied with the amount of glycerol in the mount.  Table 2 clearly shows the association between the amount of glycerol used in the mount and the time taken by the mount to dry up. Optimal dilution of glycerol prevented morphological alteration of parasitic cysts, ova, and trophozoites and conserved their internal structures, which would have been distorted on using pure glycerol due to its water extracting property.

Mount in which glycerol was used at 50% concentration could be preserved for the longest duration and also provided the best contrast when viewed under the microscope. Similar results were obtained by Vignesh et al. [9] using 0.25% iodine-glycerol combination.

## 5. Conclusion

Methylene blue-glycerol wet mount is easy to perform and stays longer; the reagents are economical and easily prepared. The procedure also aids the better detection and recognition of parasites in the stool in a peripheral laboratory, side clinic of doctors, and other laboratories in the developing countries where permanent stained smear of stool does not form a component of routine stool examination. We advocate the use of methylene blue-glycerol wet mount along with saline wet mount in a routine parasitology laboratory.

## Figures and Tables

**Table 1:** Total numbers of various intestinal parasites observed by different wet mounts techniques of faecal specimen.

Parasites	Structure observed	Various parasites among total positive samples (*N* = 50)
*Entamoeba histolytica *	Cysts and trophozoites	8
*Giardia lamblia *	Cysts	7
*Ascaris lumbricoides *	Ova	2
Hookworm	Ova	8
*Trichuris trichiura *	Ova	6
*Hymenolepis nana *	Ova	1
*Isospora belli *	Ova	6
*Cyclospora *spp.	Ova	3
*Strongyloides stercoralis *	Larvae	8
*Paramecium *spp.	Organism	1

**Table 2:** Association between the amount and time taken by glycerol mount to dry up.

Percentage of glycerol used in the solution	Time taken by the mount to dry up
10%	6 hrs
12.5%	8 hrs
16.6%	12 hrs
25%	36–48 hrs
50%	5 days
